# In Vitro Direct and Indirect Cytotoxicity Comparative Analysis of One Pre-Hydrated versus One Dried Acellular Porcine Dermal Matrix

**DOI:** 10.3390/ma15051937

**Published:** 2022-03-05

**Authors:** Renzo Guarnieri, Rodolfo Reda, Dario Di Nardo, Gabriele Miccoli, Alessio Zanza, Luca Testarelli

**Affiliations:** 1 Department of Oral and Maxillofacial Sciences, Sapienza University of Rome, 00161 Rome, Italy; renzoguarnieri@gmail.com (R.G.); rodolfo.reda@uniroma1.it (R.R.); gabriele.miccoli@uniroma1.it (G.M.); alessio.zanza@uniroma1.it (A.Z.); luca.testarelli@uniroma1.it (L.T.); 2 Private Periodontal Implant Practice, 31100 Treviso, Italy

**Keywords:** cytotoxicity, cytocompatibility, acellular matrices, porcine dermal matrices, pre-hydrated matrix, dried form matrix

## Abstract

Aim: The aim of the present study was to compare the direct and indirect cytotoxicity of a porcine dried acellular dermal matrix (PDADM) versus a porcine hydrated acellular dermal matrix (PHADM) in vitro. Both are used for periodontal and peri-implant soft tissue regeneration. Materials and methods: Two standard direct cytotoxicity tests—namely, the Trypan exclusion method (TEM) and the reagent WST-1 test (4-3-[4-iodophenyl]-2-[4-nitrophenyl]-2H-[5-tetrazolio]-1,3-benzol-desulphonated)—were performed using human primary mesenchymal stem cells (HPMSCs) seeded directly onto a PDADM and PHADM after seven days. Two standard indirect cytotoxicity tests—namely, lactate dehydrogenase (LTT) and MTT (3-[4,5-dimethyl-2-thiazolyl]-2,5-diphenyl-2H-tetrazoliumbromide)—were performed using HPMSCs cultivated in eluates from the matrices incubated for 0.16 h (10 min), 1 h, and 24 h in a serum-free cell culture medium. Results: The WST and the TEM tests revealed significantly lower direct cytotoxicity values of HPMSCs on the PHADM compared with the PDADM. The indirect cytotoxicity levels were low for both the PHADM and PDADM, peaking in short-term eluates and decreasing with longer incubation times. However, they were lower for the PHADM with a statistically significant difference (*p* < 0.005). Conclusions: The results of the current study demonstrated a different biologic behavior between the PHADM and the PDADM, with the hydrated form showing a lower direct and indirect cytotoxicity.

## 1. Introduction

Autogenous oral subepithelial connective tissue and oral epithelial tissue grafts are still considered to be the gold standard in enhancing peri-implant soft tissue regeneration and gaining attached mucosa around dental implants in the oral cavity [1]. However, autogenous tissue harvesting has important disadvantages such as increased donor site morbidity, prolonged surgical times, and withdrawal volume limits [2]. Recently, collagenous barriers of a xenogeneic origin have been introduced in peri-implant soft tissue volume augmentation procedures as an alternative to autografts. These collagen-based biomaterials are composed of type I and type III collagens and exhibit a chemotactic potential for various types of cells [3,4,5]. In addition, they have a prominent role in coagulum formation [3,6,7] and angiogenesis at wound sites [8]. Collagen-based biomaterials used in peri-implant plastic surgeries as an alternative to autografts are of a bovine and porcine origin. Porcine-derived acellular dermal matrices (PADMs) have been demonstrated to be biocompatible and possess sufficient physical, chemical, and mechanical stability to favor tissue regeneration following surgical reconstructive periodontal and peri-implant therapies [9,10]. PADMs are replaced by new connective tissue within approximately 6–9 months, resulting in a complete integration in the host tissue [11,12].

The first PADMs were commercially available in a dry form (porcine dried acellular dermal matrix, PDADM). Preclinical studies have shown that PDADMs promote the metabolic activity of human endothelial cells, oral fibroblasts, osteoblasts, and periodontal ligament cells [13,14,15,16].

In recent years, PADMs have also been processed in a hydrated form (porcine hydrated acellular dermal matrix, PHADM). Unlike the dry forms, which require a long hydration in a sterile saline solution for 20 min prior to application, PHADMs are a ready to use graft with no or minimal rehydration required prior to the implantation. Initial in vitro investigations proved that, compared with PDADMs, PHADMs could increase cell migration, adhesion, proliferation, and revascularization during early healing [17,18].

Although several studies have investigated the biologic and chemical–physical features of PADMs [2,3,4,5,6,7,8,9,10,11,12,13,14,15,16,17,18,19,20], the biocompatibility of these matrices with human primary mesenchymal stem cells (HPMSCs) is rarely described [21]. In vitro assays of HPMSCs offer a proper model for studying the interactions of these cells with matrices. The aim of this study was to compare, in vitro, the indirect and direct cytotoxicity of a PDADM versus a PHADM, both used for periodontal and peri-implant soft tissue regeneration.

## 2. Materials and Methods

The PADMs (10 × 10 mm) utilized in the current study were a porcine acellular dermal matrix supplied in a dry form (PDADM, Mucoderm; Botiss biomaterials GmbH, Zossen, Germany) and a pre-hydrated porcine acellular dermal matrix (PHADM, NovoMatrixTM Reconstructive Tissue Matrix; BioHorizons, Birmingham, AL, USA).

According to the manufacturer’s instructions for use, the PDADM received a long hydration prior to the application, which was performed in a sterile saline solution for 20 min. The PHADM, according to the manufacturer’s instructions, was “ready to use” and it required placing in a sterile lactated Ringer’s solution for just 2 min.

The HPMSCs were isolated by aspiration from the bone marrow of a 65-year-old woman that underwent surgery for medical reasons during harvesting bone from the iliac crest. The patient was treated as part of a previous approved research protocol (University Ethical Committee approbation #7413). Written consent was obtained prior to the collection of the material.

The HBMSCs were isolated and cultivated according to the steps proposed by Bartmann et al. [22]. Briefly, the concentration of cells in the heparinized human bone marrow was analyzed by an automated blood count (KX-21N, Sysmex Europe Gmbh, Bornbarch 1 22,848 Norderstedt, Germany) corresponding with 2.2 × 10^4^ cells per milliliter in a 225 cm^2^ tissue culture flask (Corning, Merk Life Science s.r.l., 93 20,149 Milano Italy). An alpha minimum essential medium (α-MEM) was supplemented with 10% fetal calf serum, 1% penicillin/streptomycin, and 2 mmol/LL-glutamine. Culturing was performed at 37 °C in a humidified atmosphere of 95% air and 5% carbon dioxide. Nonadherent cells were removed via a complete change of medium after 3 days. The medium was changed every 3 to 4 days by replacing 30% of it with a freshly supplemented medium. The cells were harvested upon reaching 70% confluency at 15 days using a solution of 0.005% trypsin and 0.02% ethylenediaminetetraacetic acid in phosphate-buffered saline. The cells were then seeded at a low density of 30 cells/mL in a 225 cm^2^ cell culture flask. The characterization of the isolated cells and the seeding of the HMSCS on the PADMs took place after two passages.

Cell Characterization

The cells were blocked with immunoglobulins and stained for 20 min to 4° using monoclonal antibodies (Beckman Coulter s.r.l., 20,060 Cassina De’ Pecchi, Milano, Italy). An HPMSC phenotyping kit (Miltenyi Biotech, Bergisch Gladbach, Germany) was used following the manufacturer’s instructions and the samples were analyzed with a MACSQuant^®^ Analyzer 10 (Miltenyi Biotech, Bergisch Gladbach, Germany). Osteogenic, adipogenic, and chondrogenic differentiation of the isolated cell populations for plastic adherence and the specific surface antigen expression patterns were analyzed according to a consensus of the International Society for Cellular Therapy (ISCT). CD105, CD73, and CD90 were positive for > 95% of the cells. CD14, CD20, CD34, and CD45 were positive for < 2% of the cells. The capability of differentiation to the osteoblasts was tested by incubation with supplemented α-MEM containing 10 nmol/L dexamethasone, 10 nmol/L beta glycerophosphate and 52 mg/L ascorbic acid. Von Kossa staining was used to detect the calcium deposits. The capability of differentiation to the adipocytes was tested after inducing incubation with supplemented α-MEM containing 1 nmol/L dexamethasone, 20 U/L insulin, 60 nmol/L indomethacin, and 0.5 mmol/L 1-methyl-3-isobutylxanthine. Adipoid droplets were confirmed through Oil Red O staining (Sigma-Aldrich, Saint Louis, MO, USA).

### 2.1. Direct Cytotoxicity Tests

A WST (4-3-(4-iodophenyl)-2-(4-nitrophenyl)-2H-(5-tetrazolio)-1,3-benzol-disulfonate) test was performed. The cells were incubated for 30 min at 37 °C to allow for cell adhesion and 2 mL of the cell culture medium was added. Seven days later, a cell proliferation reagent, WST-1 (Roche Diagnostics, catalog no. 116446807001), was used. The old medium was changed with 1000 μL of fresh medium for each well. Afterwards, 100 μL of the WST-1 reagent was added to each well, leading to a 1:10 ratio to the cell culture medium. After 4 h of incubation in a humidified atmosphere with 5% carbon dioxide at 37 °C, the medium was transferred to 96-well plates (Nunc, Germany) and absorbance was measured at 460 nm. Cells cultured on cover glasses at a density of 1 × 10^4^ cells/sample served as the controls.

The Trypan exclusion method (TEM) was then used. Trypan blue is a vital stain that allows live and dead cells to be distinguished. It is absorbed by a damaged cell membrane. A buffered isotonic salt solution with 0.4% of Trypan blue solution was prepared and 0.1 mL of cells was added. A hemacytometer (BRAND counting chamber, BLAUBRAND, Brand GMBH, Germany) and a microscope with a low magnification were used. The number of viable cells divided by the total number of cells within the grids on the hemacytometer was used to calculate the viability of the cells.

### 2.2. Indirect Cytotoxicity Tests

A lactate dehydrogenase test (LDH) was performed. The HPMSCs were seeded on 24-well cell culture plates (Nunc Germany) in 100 μL of α-MEM at a concentration of 5 × 10^3^ cells/well. After 24 h of culture in a humidified atmosphere with 5% carbon dioxide at 37 °C, the medium was removed and replaced with 150 μL (20%) eluate from the matrices (10 × 10 mm) and then incubated for 10 min, 1 h, and 24 h in a serum-free cell culture medium. Cells cultured in 1% triton X-100 in a serum-free α-MEM served as the positive controls for cytotoxicity. Cells cultured in a serum-free α-MEM served as the negative controls. After 24 h of incubation, 100 μL of the supernatant was transferred to another 24-well cell culture plate. An LDH detection kit (Roche Diagnostics, catalog no. 11644793001) was used to measure the extracellular lactate dehydrogenase (LDH) activity. The value of 490 nm was used to measure the absorbance.

An MTT test was then performed (MTT: 3-(4,5-dimethyl-2- thiazolyl)-2,5-diphenyl-2H-tetrazoliumbromide). This tetrazolium salt can be cleaved by metabolic active cells to form a formazan dye and, therefore, it indicates the number of viable cells. The HPMSCs were seeded on 24-well cell culture plates (Nunc, Germany) in 100 μL α-MEM at a concentration of 5 × 10^3^ cells/well. After 24 h of culture in a humidified atmosphere with 5% carbon dioxide at 37 °C, the medium was removed and replaced with 150 μL of eluate from the matrices (10 × 10 mm) and then incubated for 10 min, 1 h, and 24 h in a serum-free cell culture medium. Cells cultured in a serum-free α-MEM served as the positive controls. An MTT cell proliferation kit (Roche Diagnostics, catalog no. 11465007001) was used after 24 h of incubation of the cells. The value of 570 nm was used to measure the absorbance.

### 2.3. Statistical Analysis

The tests were repeated eight times per assay. The absorbance values of the test were transformed. All values of the MTT test were divided by 0.483 (control group) and the LDH test by the formula ([experiment value—low control]/[high control—low control]) × 100. For the WST test, the raw values were examined. These results were evaluated by analyses of variance for the LDH and MTT tests with the factor of time (10 min, 1 h, or 24 h) and for the WST test with the factor of material. The number of viable cells per mL of culture was measured using the formula % viable cells = [1.00 − (number of blue cells ÷ number of total cells)] × 100. The least-squared means and 95% confidence intervals were determined. A one-way analysis of variance (ANOVA) with a Tukey’s post-hoc test was used for multiple comparisons and were considered to be significant with a *p*-value < 0.05.

## 3. Results

**Figure 1:**
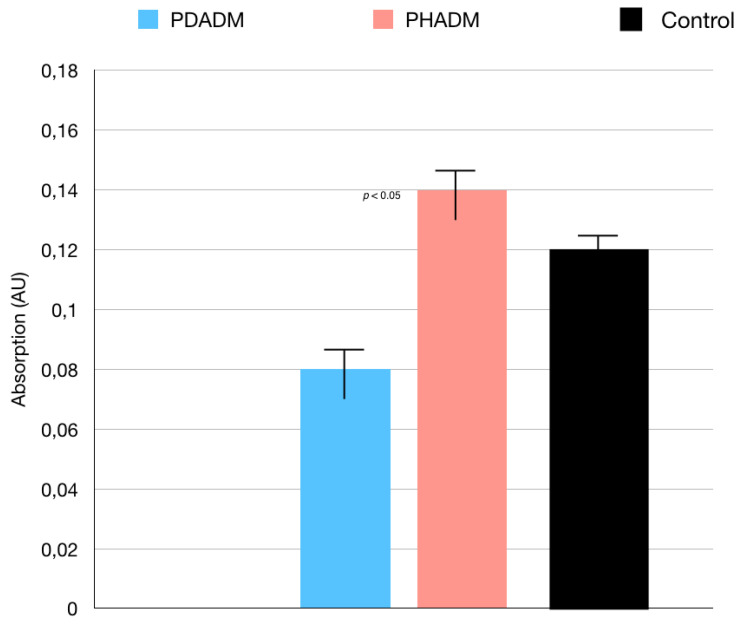
Results of the WST test of HPMSCs directly cultured onto matrices after 7 days. Cells seeded on cover glass served as controls. Each column shows the results of experiments repeated three times. (One-way analysis of variance (ANOVA) with Tukey’s post-hoc test. Values of *p* < 0.05 were considered significant.)

With regard to the direct cytotoxicity tests, the WST test results are shown in Figure 1. The test revealed that, after 7 days of culture, the direct cytotoxicity was significantly lower (*p* < 0.05) for the HPMSCs on the PHADM compared with the PDADM.

**Figure 2:**
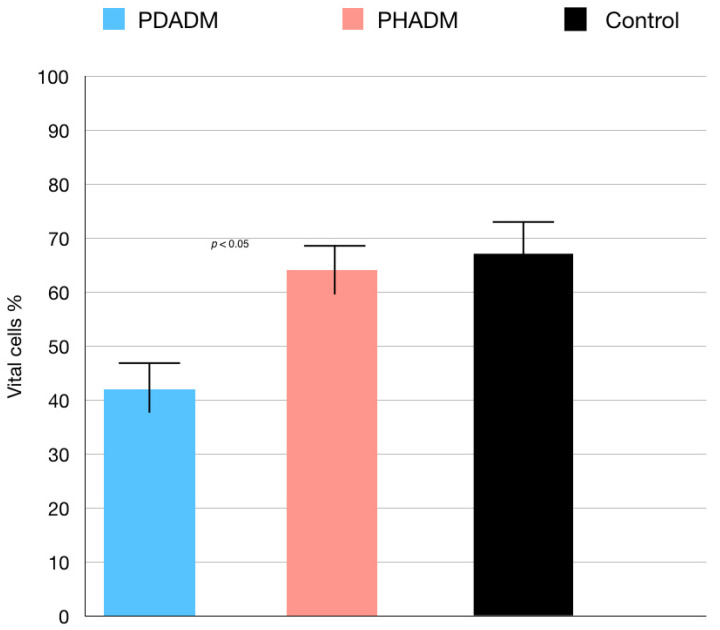
Results of the TEM of HPMSCs directly cultured on matrices after 7 days. Viable cell % = total number of viable cell per mL of aliquot/total number of cells per mL of aliquot × 100. Each column shows the results of experiments repeated three times. (One-way analysis of variance (ANOVA) with Tukey’s post-hoc test. Values of *p* < 0.05 were considered significant.)

**Figure 3:**
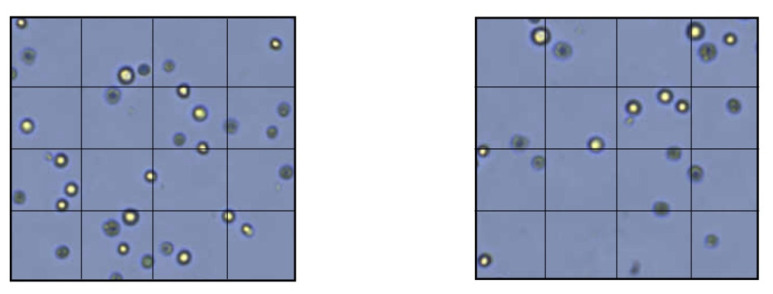
Membrane permeability test with TEM. Left: HPMSCs directly cultured onto a PDADM. Right: HPMSCs directly cultured onto a PHADM.

The results of the TEM indicating the percentage of vital cells are shown in Figure 2 and Figure 3. The test revealed that the viability of the HPMSCs on the PHADM was significantly better (*p* < 0.05) compared with the viability on the PDADM.

### Indirect Cytotoxicity Tests: LDH and MTT

**Figure 4:**
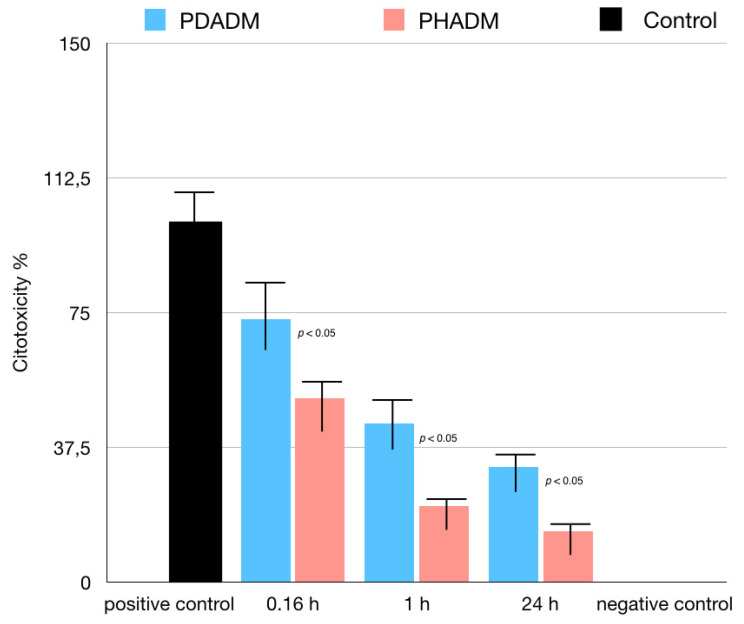
Results of LDH test of HPMSCs cultured in eluates from matrices incubated for 0.16, 1, and 24 h in serum-free cell culture medium. Cells cultured in serum-free medium served as negative controls for cytotoxicity. Cells cultured in 1% Triton X-100 in serum-free culture medium served as positive controls. Each column represents the results of the experiments repeated eight times. Cytotoxicity of eluates is shown relative to controls (least-squared means +/− 95% confidence intervals, one-way analysis of variance (ANOVA) with Tukey’s post-hoc test. Values of *p* < 0.05 were considered significant).

For the LDH test, the cells were cultured in eluates from the matrices after incubation times of 10 min, 1 h, or 24 h. The 1 h and 24 h eluates showed a moderate to low cytotoxicity in both PADMs; however, the mean values were significantly lower for the PHADM compared with the PDADM (*p* < 0.05). With an increase in the incubation time, the cytotoxicity of the eluate was significantly lower (*p* < 0.05) for both matrices and a significant statistical difference was noted between the PHADM and the PDADM (*p* < 0.05). The result of the LDH test is shown in Figure 4.

**Figure 5:**
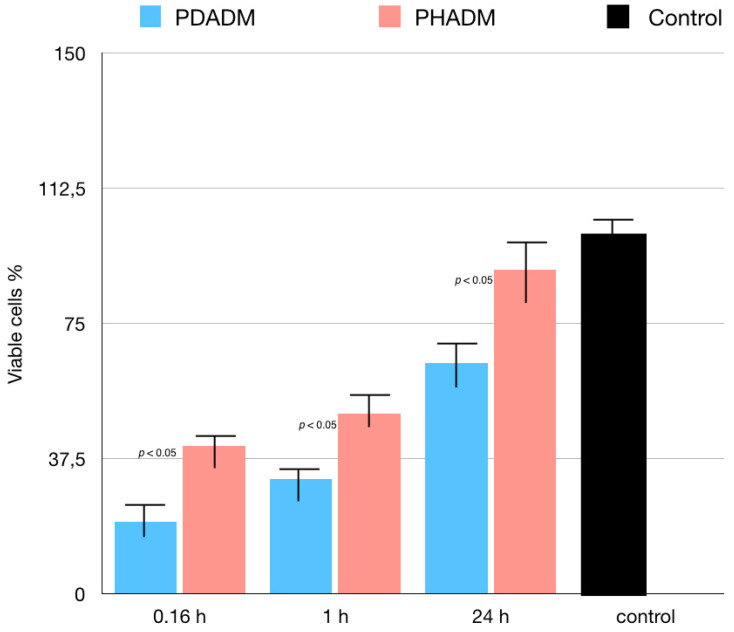
Results of MTT test of HPMSCs cultured in eluates from matrices incubated for 0.16, 1, and 24 h in serum-free cell culture medium. Cells cultured in normal serum-free culture medium served as positive controls. Viable cell % is shown relative to positive controls (least-squared means +/− 95% confidence intervals). Each column represents the results of the experiments repeated eight times. (One-way analysis of variance (ANOVA) with Tukey’s post-hoc test. Values of *p* < 0.05 were considered significant.)

The results of the MTT test performed in eluates from the matrices incubated in the serum-free medium for 10 min, 1 h, or 24 h are reported in Figure 5. The incubation time significantly influenced the proliferation of the HPMSCs in both matrices. With an increase in the incubation time, the indirect cytotoxicity was superior on the PDADM than on the PHADM (*p* < 0.05). The eluates from the PDADM after a 24 h immersion in the serum-free medium significantly enhanced the indirect cytotoxicity compared with the PDADM and the 10 min and the 1 h eluate (*p* < 0.05).

## 4. Discussion

The successful integration into the surrounding tissue, the ability to degrade and be replaced by soft connective tissue, and 3D volume stability over time are considered primary requirements of any potential device intended to be used as a replacement for autogenous connective/gingival tissue grafts. PADMs proved to possess these requirements and have previously been proposed to enhance peri-implant soft tissue regeneration, gaining attached mucosa around dental implants in the oral cavity. Several studies [2,3,4,5,6,7,8,9,10,11,12,13,14,15,16,17,18,19,20] demonstrated that due to biocompatibility as well as a sufficient physical, chemical, and mechanical stability, PADMs are replaced by new connective tissue within approximately 6–9 months, resulting in complete integration in the host tissue [9,10,11,12].

Various physical (e.g., repeated freeze-thaw cycles), chemical (e.g., treatment with a hypertonic or hypotonic solution), or enzymatic (e.g., treatment with trypsin and ethylene diamine tetra-acetic acid (EDTA)) approaches are used for the decellularization of dermal xenogeneic matrices. PADMs are commercially available in two forms: dry and pre-hydrated [23]. Unlike the dry forms, the hydrated ones require minimal rehydration prior to implantation. As the substrate structure plays an important role in the soft tissue cell behavior, in the present study we compared the direct cytotoxicity of a PDADM versus a PHADM on the HPMSCs in vitro.

In general, in vitro studies involving the membranes and matrices used in guided soft tissue regeneration are performed with fibroblasts [16,24]. Although fibroblasts share similar phenotypic features with mesenchymal stem cells, unlike as mesenchymal stem cells they do not undergo differentiation and have no colony-forming capacity [25]. In contrast, mesenchymal stem cells are quiescent most of the time but have a self-renewing capacity [26]. Moreover, mesenchymal stem cells can differentiate into multiple tissues of injured sites, including connective tissue [27]. Therefore, the present study used HPMSCs as a biological model that aimed to mimic the biological environment after wounding [28].

With a special view on the revascularization process, wound healing can be divided into three phases: the inflammation phase (from 1 h up to 14 days); the proliferation phase (from 1 day up to 14 days); and the remodeling phase (from 3 days up to 3 weeks) [29,30]. During the proliferation phase, a platelet-derived growth factor (PDGF) as a proangiogenic cytokine and growth factors such as vascular endothelial growth factor (VEGF) are released into the wound. They stimulate the sprouting angiogenesis as well as the intussusceptive angiogenesis from the pre-existing vessels [9,29,30].

The most important peculiarities of dermal matrices are not only those that trigger cell adhesion and proliferation but also those that promote revascularization, thus mimicking the integration process of the autologous connective graft [31,32]. In this study, the viability of HPMSCs was tested by WST and TEM after seven days of cell incubation; the time corresponded with the proliferative phase of wound healing. The results demonstrated an increased viability of the HPMSCs on the PHADM compared with the PDADM. This increased cell viability during the time period of the early proliferation phase supported the hypothesis that the hydrate form, compared with the dry form of the PADM, allowed a better cell adhesion and proliferation and a faster and earlier revascularization. This hypothesis was also supported by the results of recent studies by Nica et al. [19] and Lin et al. [25]. Nica et al. [19] evaluated the adsorption and release of growth factors from PHADMs versus PDADMs. PHADMs, compared with PDADMs, showed a better adsorption and growth factor release in the first 13 days. The study by Lin et al. [25] evaluated the biological behavior of primary human oral fibroblasts and periodontal ligament cells with respect to four collagen matrices, including PHADMs and PDADMs. The analysis of the expression of wound healing-related genes in two oral cell types showed that PHADMs had an increased expression of vascular endothelial growth factor-A (VEGF-A), considered by the authors to be beneficial in promoting a better revascularization of the scaffold.

In the current study, two indirect cytotoxicity tests (LDH and MTT) were performed on eluates derived from the PHADMs and PDADMs to evaluate the possible presence of volatile toxic materials washed out from these membranes. These volatile toxic materials could influence the metabolic cell activity, negatively affecting the matrix integration process and the related clinical result.

The LDH and MTT tests performed in eluates at 10 min (0.16 h), 1 h, and 24 h indicated that the longer the PHADM and the PDADM were both incubated, the greater the positive influence on the metabolic activity of the HPMSCs. Both matrices showed a greater cytotoxicity in the eluate collected after 10 min compared with those collected after 1 and 24 h. Accordingly, it may be useful to wash both the PHADM and the PDADM prior to application in order to eliminate cytotoxic substances.

However, the results of the LDH and MTT tests showed a statistically higher metabolic activity of the HPMSCs on the PHADM compared with the PDADM, which increased in all the eluates.

In the current investigation, the PHADMs and the PDADMs tested were available from the current market and prepared according to the instructions given in the manufacturers’ use sheet. The two forms of matrix, hydrated and dry, are the result of two different production processes on which a comparative discussion cannot be faced as they are unknown to the authors and owned by the manufacturers. This could represent a limitation to the discussion. It is important to point out that the objective of the present study was to examine, in vitro, the direct and indirect toxicity of these matrices toward the HPMSCs in the state in which they were placed on the market and declared ready for use. The only difference, prior to the clinical use of the two matrices, was related to hydration. The PDADM required a long hydration in a sterile saline solution for 20 min prior to the application. The PHADM required placing in a sterile lactated Ringer’s solution for just 2 min. From a clinical point of view, one might expect that, once inserted into the surgical site, the matrix is subjected to continuous hydration from the biological fluids. Consequently, the hydration process that must be carried out before using the membrane should not have a significant influence on the integration process of the same. Several studies have instead reported that the process of rehydration of dried collagen matrices contributes to the mechanical and biochemical properties of such matrices, determining a distortion on the collagen molecular conformation and an increase in the number of intramolecular hydrogen bonds [31,32,33].

Another factor that could have influenced the cell viability could be linked to the different osmotic disbalance that was created after a short or long hydration of the matrix. External mechanical forces of extracellular environments are well-recognized as playing major roles in numerous cell functions, including proliferation, differentiation, and cell–cell interactions [34]. The cytoplasmic membrane is permeable to water but is impermeable to many metabolites. When extracellular fluid osmolarity is lower than that of the intracellular fluid, cells and tissues experience hypo-osmotic stress. Conversely, hyperosmotic stress describes the situation where extracellular solute concentrations exceed those inside the cell. Osmotic imbalances between the intra- and extracellular environment affect the water flux, cell volume, and cell homeostasis. The hypo-osmotic stress causes a significant increase in the cell diameter and volume, leading to cell lysis with exposure to deionized water whereas hyperosmotic stress causes significant cell shrinkage [35]. Moreover, hypotonic shocks also induce mechanical stress on the cytoskeleton with the reorganization of actin filaments [36,37] and changes in intracellular ion concentrations that influence cell homeostasis and cellular metabolism [38].

The better biologic behavior of the hydrated samples compared with the dry samples noted in the present study could be linked to different biological properties that PADMs present following, or not following, a rehydration process. However, comparative microscopy studies are needed to confirm this assumption.

The data of the current study demonstrated a different biologic behavior between the dry and hydrated PADMs. However, it should be emphasized that although laboratory conditions allowed for a good control of the variables, a clinical setting comprises a greater structural, cellular, and tissue complexity. Therefore, performing an isolated analysis of the regeneration or stimulation potential of the matrices is more difficult in a laboratory.

## 5. Conclusions

The results of the current study demonstrated a different biologic behavior between the PHADM and the PDADM, with the hydrated form showing lower direct and indirect cytotoxicity.

## Data Availability

Data are available at renzoguarnieri@gmail.com.

## References

[1] Del Amo F.S.L., Yu S.H., Sammartino G., Sculean A., Zucchelli G., Rasperini G., Felice P., Pagni G., Iorio-Siciliano V., Grusovin M.G. (2020). Peri-implant Soft Tissue Management: Cairo Opinion Consensus Conference. Int. J. Environ. Res. Public Health.

[2] Herford A.S., Akin L., Cicciu M., Maiorana C., Boyne P.J. (2010). Use of a Porcine Collagen Matrix as an Alternative to Autogenous Tissue for Grafting Oral Soft Tissue Defects. J. Oral Maxillofac. Surg..

[3] Farndale R.W., Sixma J.J., Barnes M.J., De Groot P.G. (2004). The role of collagen in thrombosis and hemostasis. J. Thromb. Haemost..

[4] Rothamel D., Schwarz F., Sculean A., Herten M., Scherbaum W., Becker J. (2004). Biocompatibility of various collagen membranes in cultures of human PDL fibroblasts and human osteoblast-like cells. Clin. Oral Implant. Res..

[5] Thibault M.M., Hoemann C., Buschmann M. (2007). Fibronectin, Vitronectin, and Collagen I Induce Chemotaxis and Haptotaxis of Human and Rabbit Mesenchymal Stem Cells in a Standardized Transmembrane Assay. Stem Cells Dev..

[6] Kumar V.A., Taylor N.L., Jalan A.A., Hwang L.K., Wang B.K., Hartgerink J.D. (2014). A Nanostructured Synthetic Collagen Mimic for Hemostasis. Biomacromolecules.

[7] Asparuhova M.B., Stähli A., Guldener K., Sculean A. (2021). A Novel Volume-Stable Collagen Matrix Induces Changes in the Behavior of Primary Human Oral Fibroblasts, Periodontal Ligament, and Endothelial Cells. Int. J. Mol. Sci..

[8] Twardowski T., Fertala A., Orgel J., Antonio J.S. (2007). Type I Collagen and Collagen Mimetics as Angiogenesis Promoting Superpolymers. Curr. Pharm. Des..

[9] Tavelli L., McGuire M.K., Zucchelli G., Rasperini G., Feinberg S.E., Wang H.-L., Giannobile W.V. (2020). Extracellular matrix-based scaffolding technologies for periodontal and peri-implant soft tissue regeneration. J. Periodontol..

[10] Caballé-Serrano J., Zhang S., Ferrantino L., Simion M., Chappuis V., Bosshardt D.D. (2019). Tissue Response to a Porous Collagen Matrix Used for Soft Tissue Augmentation. Materials.

[11] Imber J.C., Bosshardt D.D., Stähli A., Saulacic N., Deschner J., Sculean A. (2021). Pre-clinical evaluation of the effect of a volume-stable collagen matrix on periodontal regeneration in two-wall intrabony defects. J. Clin. Periodontol..

[12] Caballé-Serrano J., Zhang S., Sculean A., Staehli A., Bosshardt D.D. (2020). Tissue Integration and Degradation of a Porous Collagen-Based Scaffold Used for Soft Tissue Augmentation. Materials.

[13] Pabst A.M., Happe A., Callaway A., Ziebart T., Stratul S.-I., Ackermann M., Konerding M.A., Willershausen B., Kasaj A. (2013). In vitro and in vivo characterization of porcine acellular dermal matrix for gingival augmentation procedures. J. Periodontal Res..

[14] Park J.S., Pabst A.M., Ackermann M., Moergel M., Jung J., Kasaj A. (2018). Biofunctionalization of Porcine-Derived Collagen Matrix Using Enamel Matrix Derivative and Platelet-Rich Fibrin: Influence on Mature Endothelial Cell Characteristics In Vitro. Clin. Oral Investig..

[15] Blatt S., Burkhardt V., Kämmerer P.W., Pabst A.M., Sagheb K., Heller M., Al-Nawas B., Schiegnitz E. (2020). Biofunctionalization of porcine-derived collagen matrices with platelet rich fibrin: Influence on angiogenesis in vitro and in vivo. Clin. Oral Investig..

[16] Lin Z., Nica C., Sculean A., Asparuhova M.B. (2021). Positive Effects of Three-Dimensional Collagen-Based Matrices on the Behavior of Osteoprogenitors. Front. Bioeng. Biotechnol..

[17] Del Amo F.S.-L., Rodriguez J.C., Asa’Ad F., Wang H.-L. (2019). Comparison of two soft tissue substitutes for the treatment of gingival recession defects: An animal histological study. J. Appl. Oral Sci..

[18] Lima R.S., Peruzzo D.C., Napimoga M.H., Saba-Chujfi E., Dos Santos-Pereira S.A., Martinez E.F. (2015). Evaluation of the Biological Behavior of Mucograft^®^ in Human Gingival Fibroblasts: An In Vitro Study. Braz. Dent. J..

[19] Nica C., Lin Z., Sculean A., Asparuhova M.B. (2020). Adsorption and Release of Growth Factors from Four Different Porcine-Derived Collagen Matrices. Materials.

[20] Vallecillo C., Toledano-Osorio M., Vallecillo-Rivas M., Toledano M., Osorio R. (2021). In Vitro Biodegradation Pattern of Collagen Matrices for Soft Tissue Augmentation. Polymers.

[21] Liu Q., Humpe A., Kletsas D., Warnke F., Becker S.T., Douglas T., Sivananthan S., Warnke P.H. (2011). Proliferation assessment of primary human mesenchymal stem cells on collagen membranes for guided bone regeneration. Int. J. Oral Maxillofac. Implants.

[22] Bartmann C., Rohde E., Schallmoser K., Pürstner P., Lanzer G., Linkesch W., Strunk D. (2007). Two steps to functional mesenchymal stromal cells for clinical application. Transfusion.

[23] Yazdanian M., Arefi A.H., Alam M., Abbasi K., Tebyaniyan H., Tahmasebi E., Ranjbar R., Seifalian A., Rahbar M. (2021). Decellularized and biological scaffolds in dental and craniofacial tissue engineering: A comprehensive overview. J. Mater. Res. Technol..

[24] Lin Z., Nica C., Sculean A., Asparuhova M.B. (2020). Enhanced Wound Healing Potential of Primary Human Oral Fibroblasts and Periodontal Ligament Cells Cultured on Four Different Porcine-Derived Collagen Matrices. Materials.

[25] Soundararajan M., Kannan S. (2018). Fibroblasts and mesenchymal stem cells: Two sides of the same coin?. J. Cell Physiol..

[26] Zupan J. (2021). Mesenchymal Stem/Stromal Cells and Fibroblasts: Their Roles in Tissue Injury and Regeneration, and Age-Related Degeneration. Fibroblasts—Advances in Inflammation, Autoimmunity and Cancer.

[27] Fu X., Liu G., Halim A., Ju Y., Luo Q., Song A.G. (2019). Mesenchymal Stem Cell Migration and Tissue Repair. Cells.

[28] Kirsner R.S., Eaglstein W.H. (1993). The wound healing process. Dermatol. Clin..

[29] Hess C.T., Kirsner R.S. (2003). Orchestrating wound healing: Assessing and preparing the wound bed. Adv. Skin Wound Care.

[30] Li J., Zhang Y.P., Kirsner R.S. (2003). Angiogenesis in wound repair: Angiogenic growth factors and the extracellular matrix. Microsc. Res. Tech..

[31] Wang A.Y., Leong S., Liang Y.-C., Huang R.C.C., Chen C.S., Yu S.M. (2008). Immobilization of growth factors on collagen scaffolds mediated by polyanionic collagen mimetic peptides and its effect on endothelial cell morphogenesis. Biomacromolecules.

[32] Kasaj A., Levin L., Stratul S.I., Götz H., Schlee M., Rütters C.B., Konerding M.A., Ackermann M., Willershausen B., Pabst A.M. (2016). The influence of various rehydration protocols on biomechanical properties of different acellular tissue matrices. Clin. Oral Investig..

[33] Bottino M.C., Jose M.V., Thomas V., Dean D.R., Janowski G.M. (2009). Freeze-dried acellular dermal matrix graft: Effects of rehydration on physical, chemical, and mechanical properties. Dent. Mater..

[34] Schwartz L., da Veiga Moreira J., Jolicoeur M. (2018). Physical forces modulate cell differentiation and proliferation processes. J. Cell Mol. Med..

[35] Roffay C., Molinard G., Kim K., Urbanska M., Andrade V., Barbarasa V., Nowak P., Mercier V., García-Calvo J., Matile S. (2021). Passive coupling of membrane tension and cell volume during active response of cells to osmosis. Proc. Natl. Acad. Sci. USA.

[36] Sinha B., Koster D., Ruez R., Gonnord P., Bastiani M., Abankwa D., Stan R.V., Butler-Browne G., Vedie B., Johannes L. (2011). Cells respond to mechanical stress by rapid disassembly of caveolae. Cell.

[37] Diz-Muñoz A., Thurley K., Chintamen S., Altschuler S.J., Wu L.F., Fletcher D.A., Weiner O.D. (2016). Membrane tension acts through PLD2 and mTORC2 to limit actin network assembly during neutrophil migration. PLoS Biol..

[38] Erickson G.R., Alexopoulos L., Guilak F. (2001). Hyper-osmotic stress induces volume change and calcium transients in chondrocytes by transmembrane, phospholipid, and G-protein pathways. J. Biomech..

